# Enhanced Frequency but Preserved Th1/Tc1 Responses of Rhinovirus-Specific T Cells in COPD

**DOI:** 10.3390/biom16101423

**Published:** 2026-10-01

**Authors:** Sara Alonso Fernandez, Raquel Reyes-Manzanas, Myriam Calle-Rubio, Juan Rodriguez-Hermosa, Esther M. Lafuente, Jesús Reiné, Pedro A. Reche

**Affiliations:** 1 Department of Immunology, Ophthalmology and ENT, Faculty of Medicine, Complutense University of Madrid, Pza Ramon y Cajal s/n, 28040 Madrid, Spain; saalon07@ucm.es (S.A.F.); raqrey02@ucm.es (R.R.-M.); melafuente@med.ucm.es (E.M.L.); 2 Pulmonology Department, Hospital Clínico San Carlos, C/Profesor Martín Lagos s/n, 28040 Madrid, Spain; mcal01@ucm.es (M.C.-R.); jlrodr01@ucm.es (J.R.-H.); 3 Department of Medicine, Faculty of Medicine, Universidad Complutense of Madrid, Pza Ramon y Cajal s/n, 28040 Madrid, Spain; 4 Oxford Vaccine Group, Department of Pediatrics, University of Oxford, Old Road Campus, Roosevelt Drive, Oxford OX3 7TY, UK

**Keywords:** COPD, rhinovirus, infection, epitope, T cells, immunopathology

## Abstract

Viral infections, particularly by rhinoviruses (RVs), represent major triggers of acute exacerbations in chronic obstructive pulmonary disease (COPD). However, the role of RV-specific T cell responses in COPD remains poorly characterized. Here, we investigated T cell recall responses to conserved RV-specific T cell epitopes in COPD patients and age-matched healthy controls using activation-induced marker and intracellular cytokine staining assays. The frequency of RV-specific CD4^+^ T cells was increased in COPD patients compared with healthy participants. RV-specific CD8^+^ T cells also tended to be increased in COPD participants, although the differences did not reach statistical significance. Functional T cell responses to RV-specific epitopes were biased toward IFN-γ and TNF-α production in COPD and healthy participants, with no significant differences between groups. Accordingly, there is a degree of functional dissociation between enhanced RV-specific CD4^+^ T cell recognition and cytokine responses in COPD. A modest inverse correlation, yet not statistically significant (*p* = 0.066), was observed between IFN-γ-producing CD4^+^ T cells and disease severity, as assessed by FEV_1_. Collectively, our findings suggest that T cell responses to conserved RV-specific epitopes are unlikely to be major drivers of COPD immunopathology, supporting their potential relevance for epitope-vaccine development in COPD.

## 1. Introduction

Chronic obstructive pulmonary disease (COPD) is a leading cause of morbidity and mortality worldwide, predominantly affecting older adults with a history of tobacco smoking, characterized by chronic inflammation leading to damage of the airways and lung parenchyma [1,2]. In this sense, COPD has classically been associated with a predominantly Th1 (CD4^+^ T cell helper type 1)/Tc1 (cytotoxic CD8^+^ T cell) inflammatory profile characterized by increased interferon-gamma (IFN-γ) and tumor necrosis factor-alpha (TNF-α) [3]. However, there is increasing evidence supporting a contribution from Th17-mediated inflammation in COPD [4,5]. Furthermore, eosinophilia and Th2-cytokines have also been reported in a subset of COPD patients (eosinophilic COPD) [6,7], highlighting the complexity of the disease.

COPD is also characterized by repeated exacerbations, often triggered by environmental stimuli, including infections, that contribute to progressive lung function deterioration [8,9]. In particular, viral respiratory infections, especially those caused by rhinoviruses (RVs), are major culprits of COPD exacerbations and disease progression [10,11,12,13,14,15,16]. Indeed, RVs replicate in the respiratory mucosa and cause most of the upper respiratory tract infections in humans, including the common cold [17,18]. RVs display remarkable genetic diversity. Over 170 antigenically distinct serotypes of RVs have been identified and classified into three different species: RV-A, RV-B and RV-C [19]. This diversity is particularly high in the capsid protein VP1, which facilitates viral entry through interactions with cell surface receptors, including ICAM-1 (CD54) for RV-A and B [20,21,22] and CDHR3 for RV-C [20,23]. The most prevalent RV species are RV-A and RV-C, which are also responsible for most COPD exacerbations, particularly RV-C [14,17,24,25]. Moreover, RV infections frequently predispose patients to secondary bacterial infections, further enhancing COPD exacerbations [26].

The mechanism by which RV infections lead to COPD exacerbations is not completely understood but host–virus interactions and antiviral immunity play a relevant role. Studies have revealed that COPD patients display aberrant epithelium differentiation, which may facilitate RV infections [27]. Moreover, immune responses in patients with COPD often exhibit altered type I interferon responses, which lead to impaired antiviral immunity and prolonged inflammatory responses [28,29,30]. Features of T cell dysfunction, such as T cell exhaustion, have also been reported in COPD [31,32]. In parallel, it has been shown that RV infection prompts circulating antigen-specific T cells to move into the lung, where they accumulate following repeated infections and may contribute to airway inflammation [33]. Indeed, in healthy individuals, RV-specific T cells display a typical Th1 phenotype, characterized by the production of inflammatory cytokines such as IFN-γ, interleukin-2 (IL-2) and TNF-α [34,35,36]. However, RV-specific T cell responses in COPD patients remain poorly characterized. A better understanding of these responses is important for elucidating their role in COPD and for informing the development of vaccines against RV.

In the present exploratory study, we compared T cell recall responses in COPD patients and age-matched healthy controls to peptide pools, RV-M4 and RV-M8, consisting of conserved RV-specific CD4^+^ and CD8^+^ T cell epitopes, respectively [37,38]. To this end, peripheral blood mononuclear cells (PBMCs) were stimulated with the relevant peptide pools and T cell activation was assessed through activation-induced marker (AIM) assays and cytokine responses by intracellular cytokine staining (ICS) assays. We observed that the frequency of antigen-specific CD4^+^ T cells responding to RV-specific epitopes was increased in COPD participants compared with healthy controls. However, cytokine responses to the same epitopes displayed a predominantly Th1 profile and were comparable in both groups, suggesting some functional dissociation between enhanced RV-specific CD4^+^ T cell recognition and cytokine production in COPD. In contrast, the frequency of IFN-γ and TNF-α-producing CD8^+^ T cells tended to be increased in COPD participants compared with healthy controls, concomitant with the frequency of antigen-specific CD8^+^ T cells, although these increases did not reach statistical significance. Furthermore, we observed a modest inverse correlation between the frequency of IFN-γ-producing CD4^+^ T cells and COPD severity. Collectively, these findings indicate that T cell responses to the selected RV-specific epitopes are preserved but not exaggerated in COPD patients and may contribute to antiviral immunity without a substantial immunopathological contribution in COPD exacerbations. These observations support the potential utility of these epitopes for vaccine development in COPD.

## 2. Materials and Methods

### 2.1. Participants

A total of 44 participants aged 48 to 77 years, including 23 COPD patients and 21 age-matched healthy individuals, were recruited at the Hospital Clínico San Carlos, Madrid, Spain. The sample size was intended to provide foundational insights into RV-specific immune responses in COPD. Demographic and clinical data are described in Table 1. Eligibility and inclusion/exclusion criteria for participants were previously described in the published protocol of the study [39]. COPD patients met the spirometry criteria for COPD: Forced Expiratory Volume in 1 s (FEV_1_)/Forced Vital Capacity (FVC) < 70%. Exclusion criteria included being underage, having known systemic inflammatory or autoimmune diseases and/or ongoing immunosuppressive therapy. The study protocol was approved by the Ethics Committee of the Hospital Clínico San Carlos, Madrid, Spain (23/799-E) and was conducted in accordance with the Declaration of Helsinki. Written informed consent was obtained from all participants prior to enrollment. Participant confidentiality was maintained throughout the study, and all data were anonymized and securely stored.

### 2.2. Sample Collection and Processing

Blood samples were collected from participants in three Vacutainer tubes (9 mL each) with EDTA (BD Biosciences, Franklin Lakes, NJ, USA). Peripheral blood mononuclear cells (PBMCs) were isolated from blood samples by density gradient separation with Ficoll-Paque^TM^ Plus (Thermo Fisher Scientific, Waltham, MA, USA) as described elsewhere [40]. PBMCs within the interface layer were carefully collected, washed twice with cold phosphate-buffered saline (PBS) by centrifugation at 300 g for 5 min. Subsequently, cells were resuspended in RPMI 1640 medium (Gibco, Waltham, MA, USA) supplemented with 5% heat-inactivated human serum (Gibco, Waltham, MA, USA), 2 mM L-glutamine (Lonza, Basel, Switzerland), 100 U/mL penicillin (Lonza, Waltham, MA, USA) and 100 μg/mL streptomycin (Lonza, Waltham, MA, USA) (RPMI complete medium). Cells were counted using Trypan blue dye in a Neubauer chamber under light microscope. About 10^6^ PBMCs were obtained per mL of blood. Freshly isolated PBMCs were used in all the experiments.

### 2.3. RV-Specific T Cell Epitopes and Peptide Pools

Peptides corresponding to conserved RV-specific CD8^+^ and CD4^+^ T cell epitopes were synthesized at >95% purity (GenScript, Rijswijk, Netherlands). The CD4 epitope set comprised three conserved peptides from RV-A and four from RV-C, targeting various HLA II molecules [37]. Similarly, the CD8 epitope set comprised five conserved epitopes from RV-A and four from RV-C, targeting multiple HLA class I molecules [38]. Lyophilized peptides were resuspended in 40% DMSO and two peptide pools were generated: (i) RV-M4, containing the CD4 epitope set and (ii) RV-M8, containing the CD8 epitope set. Peptide pool stock solutions were made at 0.5 mM (each peptide) in 40% DMSO. Peptide pools were used at a concentration of 5 µM/peptide and DMSO ≤ 0.4%. Epitopes and peptide pools are described in Supplementary Table S1.

### 2.4. Immunophenotyping Assays

Baseline lymphocytes, B cells and various CD8^+^ and CD4^+^ T cell populations, including naive, memory and follicular T cells, were determined without any stimulation in all participants. Freshly isolated PBMCs were washed with PBS and stained protected from the light with Live/Dead Fixable Violet (Thermo Fisher Scientific, Waltham, MA, USA) for 15 min at 4 °C, and then for another 15 min with a cocktail of antibodies including anti-human CD19/CD20 (HIB19/2H7, PE/Cy7, Biolegend, San Diego, CA, USA), anti-human CD3 (SK7, APC-Cy7, Biolegend, San Diego, CA, USA), anti-human CD4 (SK3, BV605, Biolegend, San Diego, CA, USA), anti-human CD8 (SK1, BV785, Biolegend, San Diego, CA, USA), anti-human CD185 (J252D4, APC, Biolegend, San Diego, CA, USA) and anti-human CD45RA (HI100, PE, Biolegend, San Diego, CA, USA) antibodies. Cell samples were analyzed by flow cytometry.

### 2.5. Activation-Induced Marker (AIM) Assay

AIM assays were aimed at determining peptide-induced expression of CD69, OX40 (CD134), CD40L (CD154) and 4-1BB (CD137) in T cells. To this end, PBMCs were cultured in 96-well round-bottom plates (1 × 10^6^ cells per well) in RPMI complete medium (150 µL per well) for 24 h at 37 °C and 5% CO_2_ in the presence of RV peptide pools (RV-M4 or RV-M8) at a final concentration of 5 µM/peptide. Previously, cells were blocked with 0.5 µg/mL of anti-CD40 monoclonal antibody (Miltenyi Biotec, Bergisch Gladbach, Germany) for 15 min at room temperature. As positive control, cells were stimulated with anti-Biotin MACSiBead^TM^ particles and biotinylated antibodies against human CD2, CD3 and CD28 (Miltenyi Biotec, Bergisch Gladbach, Germany). As negative control, PBMCs were cultured with RPMI complete medium including 0.4% DMSO (mock). After culture, cells were harvested from the plates, washed with PBS, and stained, first with Live/Dead Fixable Violet (Thermo Fisher Scientific, Waltham, MA, USA) and then with the antibodies indicated in Supplementary Table S2 for surface markers defining T cell lineage and activation. Stains were carried out by incubating the cells, protected from the light, with the relevant reagents/antibodies for 15 min at 4 °C. Finally, cell samples were analyzed by flow cytometry to detect activated T cell populations.

### 2.6. Detection of Cytokine Production by T Cells

IFN-γ, TNF-α, IL-2, IL-4 and IL-10 production by CD4^+^ and CD8^+^ T cells was determined by intracellular cytokine staining (ICS) assays. PBMCs in RPMI complete medium were incubated in 96-well round-bottom plates (1 × 10^6^ PBMCs per well) at 37 °C and 5% CO_2_ for 24 h in the presence of RV peptide pools (RV-M4 or RV-M8) at a final concentration of 5 µM/peptide. As positive control, cells were incubated with a combination of antibodies against human CD2, CD3 and CD28 (Miltenyi Biotec, Bergisch Gladbach, Germany). As negative control, PBMCs were incubated with 0.4% DMSO in RPMI complete medium (mock). Brefeldin A (GolgiPlug^TM^, BD Biosciences, Franklin Lakes, NJ, USA) and monensin (GolgiStop^TM^, BD Biosciences, Franklin Lakes, NJ, USA) were added four hours after stimulation. The following day, cells were harvested, washed twice and stained with Live/Dead Fixable Violet (Thermo Fisher Scientific, Waltham, MA, USA) and then stained with antibodies against surface markers defining T cell lineage (see Supplementary Table S3). Both incubations were carried out for 15 min each, protected from the light, with the relevant reagents/antibodies at 4 °C. After the extracellular staining, cells were washed twice in cold PBS and fixed/permeabilized using CytoFix/Cytoperm^TM^ (BD Biosciences, Franklin Lakes, NJ, USA) following the manufacturer’s instructions. Subsequently, cells were incubated in the dark with anti-IFN-γ, anti-TNF-α, anti-IL-2, anti-IL-4, and anti-IL-10 antibodies (see Supplementary Table S3) for intracellular staining for 30 min at 4 °C. Finally, cells were analyzed by flow cytometry to detect cytokine-producing T cells.

### 2.7. Flow Cytometry Analysis

All samples were acquired on a BD Celesta cytometer (BD Biosciences, Franklin Lakes, NJ, USA) and analyzed with FlowJo^TM^ software version 10 (Tree Star, Ashland, OR, USA). Prior to acquisition, cell samples were washed in cold PBS by centrifugation at 300 g for 5 min and resuspended in PBS with 1 mM EDTA (300 μL of final volume/sample). For cell data analysis of PBMCs, lymphocytes were selected on forward scatter (FSC) and side scatter (SSC) and subsequently gated on the relevant surface markers. Positive gates for intracellular cytokine staining were defined using Fluorescence Minus One (FMO) controls (Supplementary Figure S1). Compensation matrices were set using compensation beads (BD Biosciences, Franklin Lakes, NJ, USA) and ArC™ Amine Reactive Compensation beads (Thermo Fisher Scientific, Waltham, MA, USA).

### 2.8. Statistical Analysis

Statistical analyses were performed using GraphPad Prism 8 (GraphPad Software Inc., San Diego, CA, USA). Non-parametric Mann–Whitney U tests were conducted to compare T cell populations between COPD and healthy participants. The relationship between antigen-specific CD4^+^ and CD8^+^ T cell populations responding to RV peptide pools was assessed using Spearman’s correlations. Spearman’s correlations were also used to identify associations between peptide-induced T cell responses and lung function parameters in COPD. All statistical tests were two-sided, and *p* < 0.05 was considered significant. Given the exploratory nature of the analysis, unadjusted *p*-values were considered without correction for multiple comparisons.

## 3. Results

### 3.1. Features of Selected RV-Specific T Cell Epitopes

To characterize RV-specific T cell responses, we assembled two peptide pools (RV-M4 and RV-M8) comprising conserved T cell epitopes from RV-A and RV-C that display promiscuous binding to human leukocyte antigen (HLA) molecules (Supplementary Table S1). Briefly, the peptide pool RV-M4 includes seven CD4^+^ T cell epitopes (three from RV-A and four from RV-C), which can be recognized by the majority of the population, as they can bind to numerous HLA class II molecules [37]. RV-M8 consists of nine CD8^+^ T cell epitopes (five from RV-A and four from RV-C), which can bind to various HLA class I molecules and are the only RV-specific CD8^+^ T cell epitopes known to date [38]. Peptides in RV-M4 range from 12 to 18 residues and are mostly from viral capsid proteins (four out of seven), three from VP4 (RV-A) and one from VP2 (RV-C). In contrast, peptides in RV-M8 are mostly derived from the non-structural RNA-dependent RNA polymerase 3D (six out of nine). All peptides in RV-M8 are nine amino acids in length, except for a 16-mer peptide (GLEPLDLNTSAGFPYV) derived from RV-C. This peptide can bind to A*02:01 and induce A*02:01-restricted cytotoxic CD8^+^ T cell responses [38]. Moreover, it can also elicit CD4^+^ T cell responses [37], and so it is also included in the peptide pool RV-M4.

### 3.2. Clinical and Immunological Features of COPD Participants

**Figure 1:**
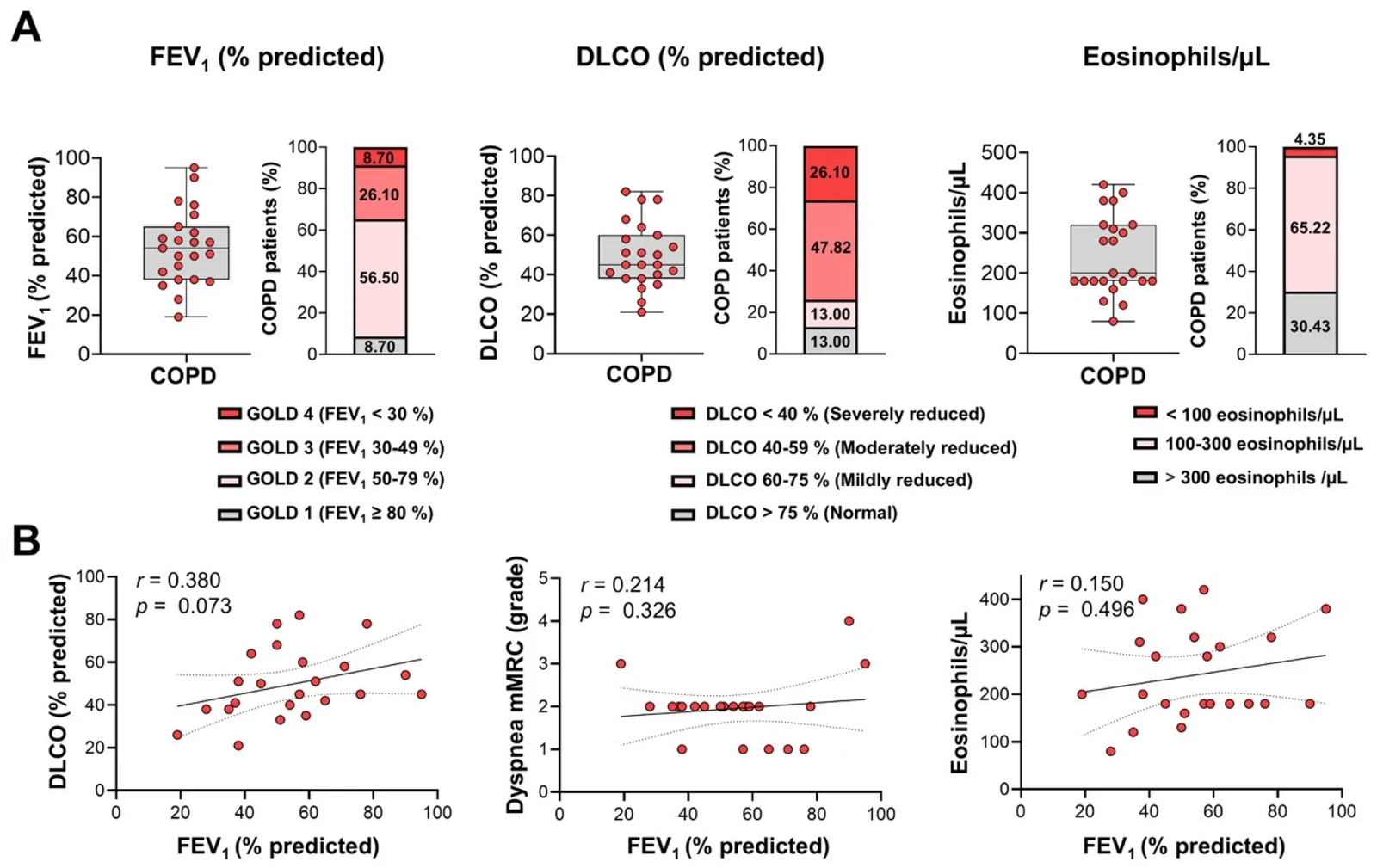
Clinical parameters of COPD participants. (**A**) FEV_1_ (% predicted), DLCO (% predicted) and eosinophils/µL in COPD participants (*n* = 23) are represented as box plots. Bar columns along the box plot show the percentage of COPD participants in clinically relevant ranges. (**B**) Graphs depicting correlations between DLCO (% predicted), grade of Dyspnea mMRC and eosinophils/µL with FEV_1_ (% predicted) in COPD patients. The solid and dotted lines represent linear regressions and 95% confidence intervals, respectively. Spearman’s correlation coefficients (*r*) and the corresponding *p*-values are indicated.

We recruited 23 participants diagnosed with COPD and 21 healthy individuals, with a balanced distribution in terms of age, between 48 and 77 years of age, and Body Mass Index (BMI). Demographic and clinical data are summarized in Table 1. COPD participants were predominantly women (18 out of 23), while healthy participants included 12 women and 9 men. Triple therapy was the most prescribed maintenance therapy in COPD patients (60.87%). According to the Global Initiative for Chronic Obstructive Lung Disease (GOLD), which classifies COPD severity from GOLD 1 (mild) to GOLD 4 (very severe) based on post-bronchodilator forced expiratory volume in 1 s (FEV_1_) (% predicted) [41], the majority of COPD patients (56.50%) were classified as GOLD 2 (Figure 1A). Similarly, the majority of patients (47.82%) had moderately reduced diffusing capacity of the lungs for carbon monoxide (DLCO) (% predicted), with values ranging from 40 to 59 counts below 300 cells/µL in the majority of patients (~70%); additionally, none had counts above 500 cells/µL, a threshold commonly considered indicative of eosinophilia [42]. Nonetheless, an eosinophilic COPD component was likely present within the cohort, as 30.4% of participants exhibited blood eosinophil counts >300 cells/µL [6,7], which is also predictive of responsiveness to inhaled corticosteroids [43].

Given the variability of COPD participants, we also explored the relationship between FEV_1_ (% predicted) and three clinical parameters: DLCO (% predicted), dyspnea and eosinophil count, using Spearman’s correlations. As shown in Figure 1B, there is a modest correlation in our COPD cohort between FEV_1_ (% predicted) and DLCO (% predicted), although this association did not reach statistical significance (*r* = 0.380, *p* = 0.073). The correlations between FEV_1_ (% predicted) and dyspnea, as well as between FEV_1_ (% predicted) and eosinophil counts, were weaker and less significant.

**Figure 2:**
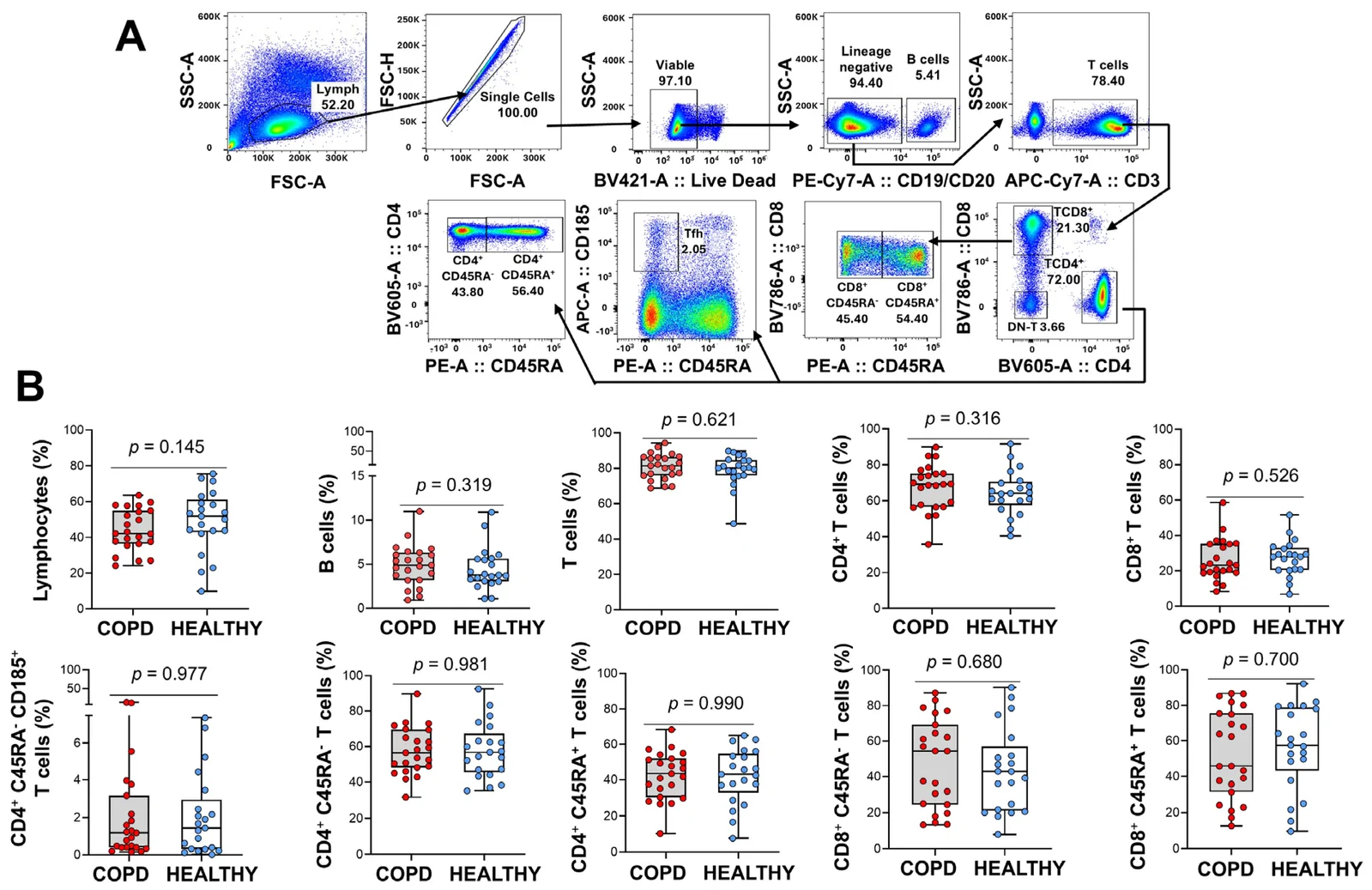
Immunophenotypic comparison of the cohort. Distinct lymphocyte populations present in PBMCs from COPD to healthy age-matched participants were analyzed by flow cytometry prior to peptide stimulation. Cell populations included total lymphocytes, B cells, T cells (CD8^+^ and CD4^+^), follicular CD4^+^ T cells (Tfh), CD45RA^+^ (naive-enriched) and CD45RA^-^ (memory-enriched) CD8^+^ and CD4^+^ T cells. (**A**) Gating strategy of PBMCs for one representative COPD patient. The following gates were applied: (1) selection of lymphocytes using the light scatter parameters (FSC-A and SSC-A), (2) exclusion of doublets and selection of singlets, (3) selection of the viable lymphocytes, (4) identification of non-B cell (lineage negative) and B cell populations, (5) identification of T cell population (CD3^+^), (6) identification of CD4^+^ and CD8^+^ T cell subsets, (7) identification of Tfh cells within CD4^+^ T cells (CD45RA^-^ CD185^+^) and (8) identification of CD45RA^+^ and CD45RA^-^ CD4^+^ and CD8^+^ T cells. (**B**) Box plots showing the frequency of the different cell populations (%) in COPD (red) (*n* = 23) and healthy (blue) (*n* = 21) participants. Statistically significant differences were calculated using non-parametric two-tailed Mann–Whitney U tests. *p*-values are shown.

We also characterized the baseline immune status in our study population. We specifically determined in non-stimulated freshly isolated PBMCs the frequency of lymphocytes, B cells and major T cell subsets, including CD4^+^ and CD8^+^ T cells, CD45RA^+^ (naive-enriched) and CD45RA^-^ (memory-enriched) CD4^+^ and CD8^+^ T cells, and follicular helper T (Tfh) cells, defined as CD4^+^CD45RA^-^CD185^+^ T cells (Figure 2A). As shown in Figure 2B, no statistically significant differences were detected in the frequency of the distinct cell populations between COPD patients and age-matched healthy individuals (Figure 2B). However, the frequency of immune cell populations in COPD patients was often more variable than in healthy participants. Moreover, a reduction in the frequency of lymphocytes and CD8^+^ T cells, particularly in naive CD8^+^ T cells, was apparent in COPD patients compared with healthy participants. Conversely, memory (CD45RA^-^) CD8^+^ T cells tended to be higher in COPD than in healthy participants. Very few Tfh cells were detected in both COPD and healthy individuals. A modest increase in the frequency of CD4^+^ T cells could also be observed in COPD participants. However, the frequency of memory and naive CD4^+^ T cell populations was comparable between COPD and healthy participants.

### 3.3. Antigen-Specific T Cells Recognizing Conserved RV-Specific Epitopes Are Increased in COPD

**Figure 3:**
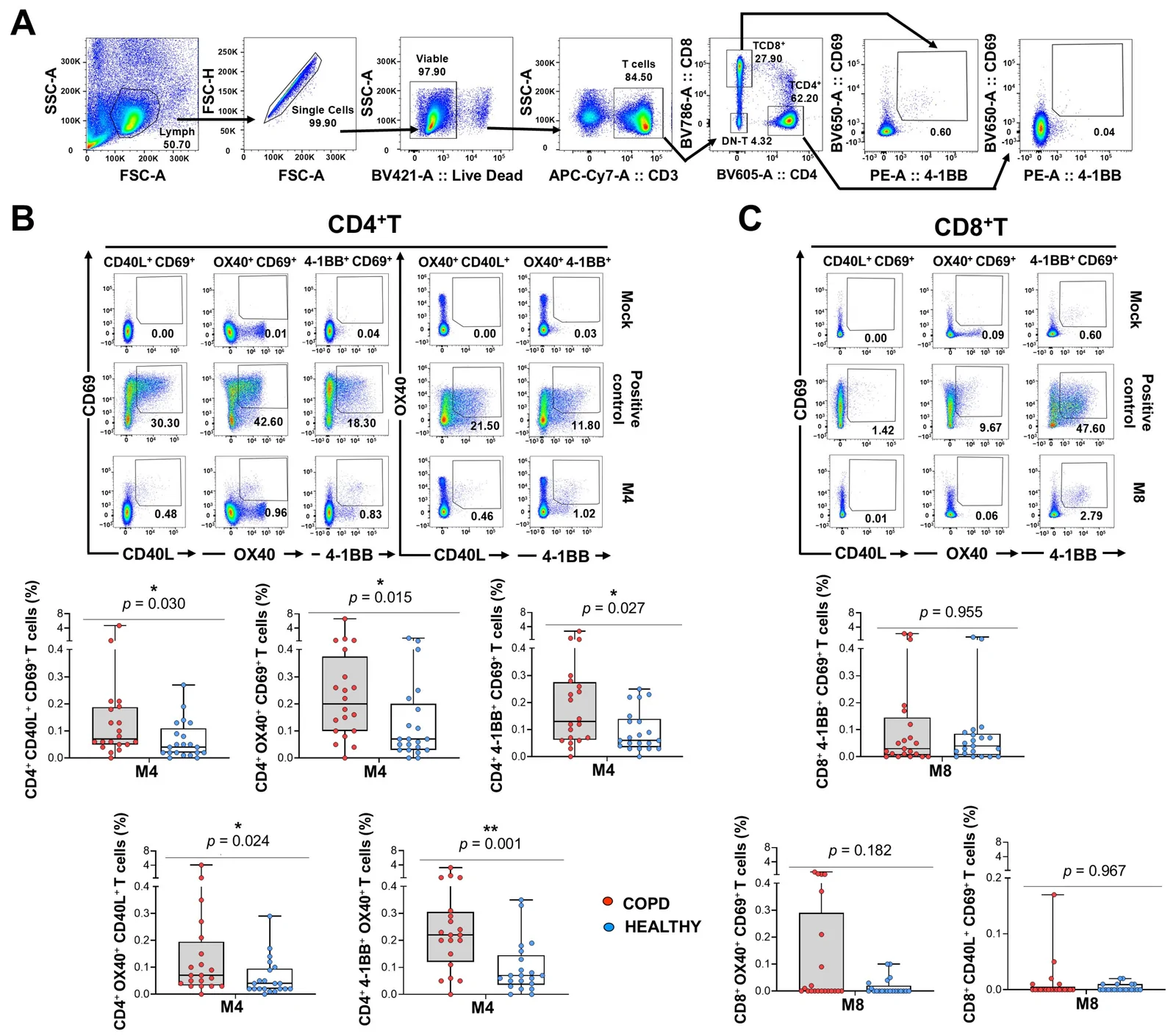
T cell activation by conserved RV-specific epitopes in COPD and healthy participants. PBMCs from COPD and healthy age-matched individuals were stimulated with peptide pools RV-M4 (M4) and RV-M8 (M8) for 24 h and T cell activation was determined by AIM assays. As a negative control, cells were cultured in RPMI complete medium with DMSO (0.4%) (mock condition) and as a positive control cells were stimulated using antibodies anti-CD2/CD3/CD28. (**A**) Gating strategy used to analyze activated T cells. A representative gating strategy for 4-1BB^+^CD69^+^ CD8^+^ and CD4^+^ T cells in unstimulated cells (mock condition) is shown: (1) lymphocytes were first selected using the light scatter parameters (FSC-A and SSC-A), (2) singlet selection, (3) selection of viable lymphocytes, (4) selection of T cells (CD3^+^), (5) selection of CD4^+^ and CD8^+^ T cell subsets and (6) selection of 4-1BB^+^CD69^+^ cells in CD8^+^ and CD4^+^ T cells. (**B**) Activated CD4^+^ T cells. Representative dot plots (mock, positive control and M4 stimulation) depicting CD4^+^ T cells co-expressing CD69 together with CD40L, OX40 and 4-1BB along with CD4^+^ T cells co-expressing OX40 together with CD40L and 4-1BB in a selected COPD participant are shown at the top. The frequency in percentage of activated CD4^+^ T cell subsets obtained in COPD and healthy participants in response to RV-M4 is shown at the bottom, represented as box plots. (**C**) Activated CD8^+^ T cells. Representative dot plots (mock, positive control and M8 stimulation) depicting CD8^+^ T cells co-expressing CD69 together with CD40L, OX40, and 4-1BB in a selected COPD participant are shown at the top. The percentage of activated CD8^+^ T cell subsets obtained in COPD and healthy participants in response to RV-M8 is shown below, represented as box plots. In panels (**B**,**C**), the values represented in the graphs (% of activated T cell subsets responding to RV peptide pools) were obtained after subtracting the corresponding values in unstimulated cells (Mock). Negative values resulting after subtractions were assigned to 0. Red data points are COPD patients (*n* = 21) and blue points represent healthy subjects (*n* = 21). The AIM assay was performed in 21 out of 23 COPD participants due to a lack of sufficient cells from two of the participants. Statistically significant differences were obtained by applying two-tailed Mann–Whitney U tests to compare peptide-responding activated T cell populations between COPD patients and healthy individuals, and *p*-values are shown. Statistically significant *p*-values < 0.05 and <0.01 are labeled with * and **, respectively.

We used activation-induced marker (AIM) assays to evaluate T cell activation responses to RV-specific epitopes (details in Materials and Methods). Briefly, PBMCs from COPD patients and healthy controls were cultured *in vitro* with conserved RV peptide pools: RV-M4 to stimulate CD4^+^ T cells and RV-M8 to stimulate CD8^+^ T cells (5 µM of peptides in RPMI complete medium for 24 h). As positive control, cells were stimulated with antibodies anti-CD2/CD3/CD28. As negative control, cells were cultured with 0.4% DMSO in RPMI complete medium (mock condition). After stimulation, cells were stained with an AIM panel and analyzed by flow cytometry (Figure 3A). Frequency of AIM^+^ T cells responding to peptides was obtained after subtracting the mock condition. For CD4^+^ T cells, we paid particular attention to cells co-expressing CD40L and OX40, or OX40 and 4-1BB, since they are considered to define antigen-specific CD4^+^ T cell subpopulations [44,45]. In addition, we also analyzed activated CD4^+^ T cells co-expressing CD69 together with OX40, CD40L or 4-1BB. These same combinations of markers were also used to analyze activated CD8^+^ T cells, although antigen-specific CD8^+^ T cells are primarily defined by the co-expression of CD69 and 4-1BB [46]. As shown in Figure 3B, COPD patients exhibited a significantly higher frequency of CD69^+^ CD4^+^ T cells compared with healthy controls, responding to RV-specific epitopes (peptide pool RV-M4). Similarly, the frequency of antigen-specific CD4^+^ T cells was also significantly enhanced in COPD participants. Indeed, the most significant change between COPD and healthy participants occurred in antigen-specific CD4^+^ T cells co-expressing OX40 and 4-1BB (*p* < 0.001). COPD participants also tended to have higher frequencies of RV-M8-responsive antigen-specific (4-1BB^+^CD69^+^) CD8^+^ T cells than healthy controls, although the differences did not reach statistical significance (Figure 3C). Similarly, other subsets of CD69^+^ CD8^+^ T cells also tended to be increased in COPD patients (Figure 3C) but without reaching statistical significance.

**Figure 4:**
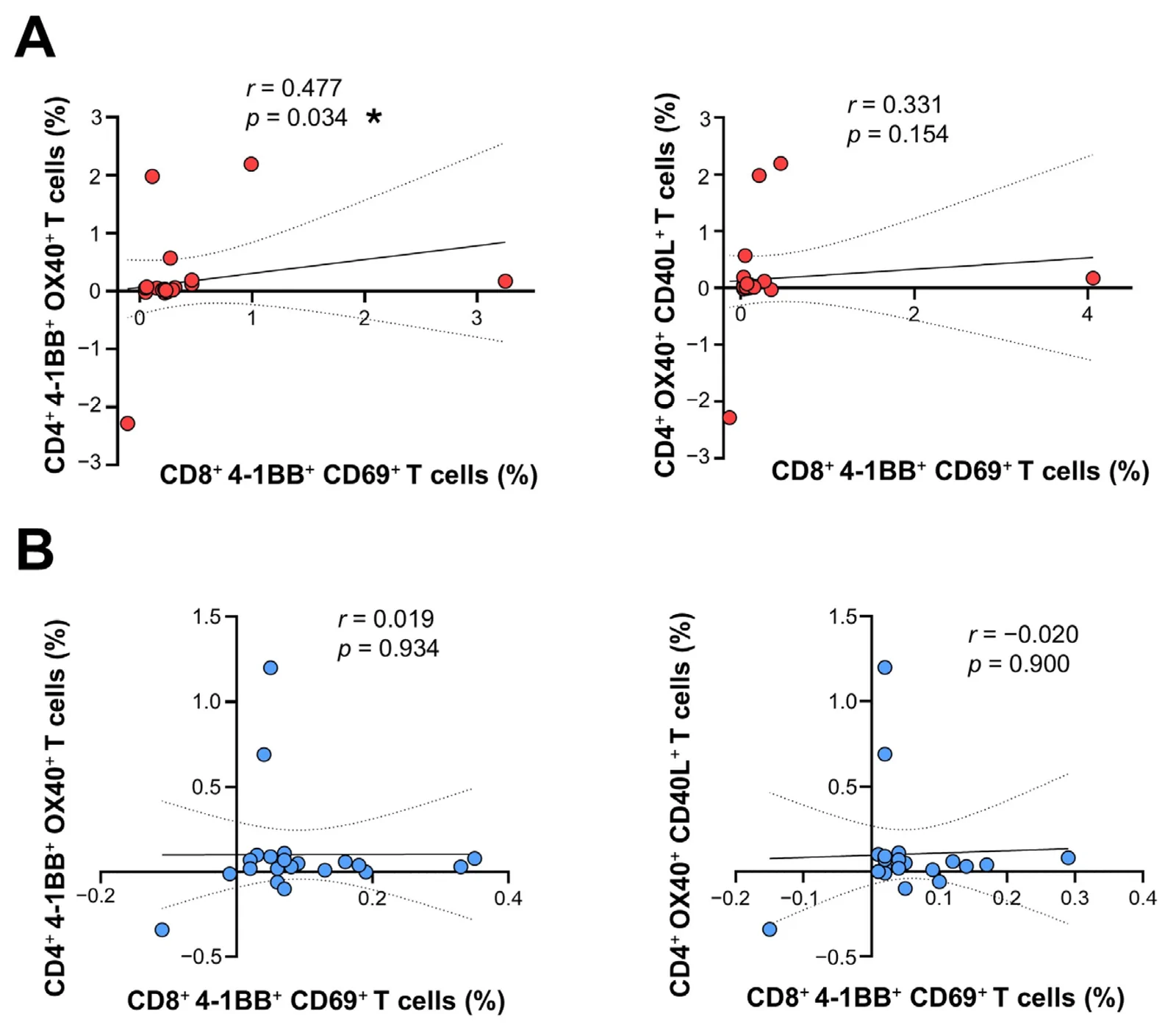
Correlation between antigen-specific CD4^+^ and CD8^+^ T cells responding to RV-specific epitopes. Spearman’s correlations between the frequency of antigen-specific CD4^+^ and CD8^+^ T cells responding to RV peptide pools in COPD (**A**) and healthy participants (**B**). Antigen-specific CD4^+^ T cells were those responding to peptide pool RV-M4 and characterized by the co-expression of OX40 together with 4-1BB or CD40L. Antigen-specific CD8^+^ T cells were those responding to peptide pool RV-M8 and characterized by the co-expression of CD69 and 4-1BB. The solid and dashed lines depict linear regressions and 95% confidence intervals, respectively. Spearman’s correlation coefficient (*r*) and the corresponding *p*-value are indicated. * *p* < 0.05, considered statistically significant.

We also identified a modest correlation between the frequency of antigen-specific OX40^+^4-1BB^+^ and OX40^+^CD40L^+^ CD4^+^ T cells and that of antigen-specific CD8^+^ T cells in COPD participants, as determined by Spearman’s correlations (*r* = 0.477, *p* = 0.034 and *r* = 0.331, *p* = 0.154, respectively) (Figure 4A). In contrast, no correlation was found between antigen-specific CD4^+^ and CD8^+^ T cells in healthy participants (Figure 4B). These findings suggest a coordinated RV-specific T cell response in COPD.

### 3.4. COPD Participants Displayed Contained Th1/Tc1 Cytokine Responses to Conserved RV-Specific Epitopes

**Figure 5:**
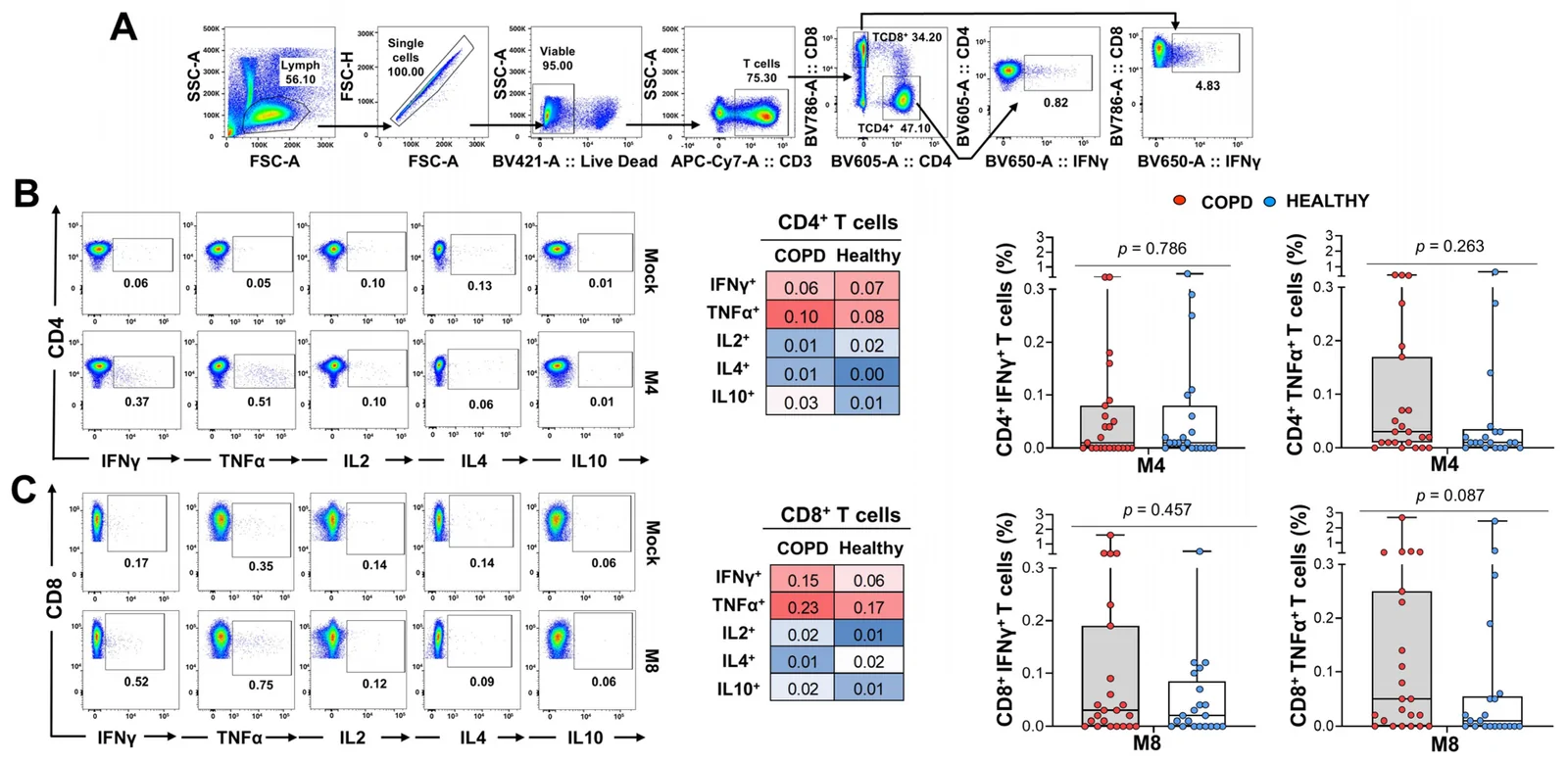
T cell cytokine responses to conserved RV-specific epitopes in COPD and healthy participants. Cytokine-producing T cells (IFN-γ, TNF-α, IL-2, IL-4 and IL-10) were determined by intracellular cytokine staining assays in PBMCs from COPD and healthy participants stimulated with peptide pools RV-M4 (M4) or RV-M8 (M8). As a negative control, cells were cultured in RPMI complete medium with 0.4% DMSO (mock). As a positive control, cells were stimulated with antibodies anti-CD2/CD3/CD28. Cytokine responses (frequency of cytokine-producing cells) to peptide pools were determined after subtracting the corresponding data from the mock condition. Negative values after subtraction were assigned to 0. (**A**) Gating strategy used to analyze cytokine production in CD8^+^ and CD4^+^ T cell subsets, exemplified with IFN-γ on PBMCs stimulated with antibodies anti-CD2/CD3/CD28. Positive gates for cytokine-producing T cells were obtained for each cytokine after FMO controls (Supplementary Figure S1). Cytokine-producing CD4^+^ and CD8^+^ T cells responding to RV peptide pools are shown in panels (**B**,**C**), respectively. The panels on the left show representative dot plots of cytokine-producing CD4^+^ and CD8^+^ T cells in response to the peptide pool RV-M4 (M4) and RV-M8 (M8), respectively, and a mock of a selected COPD participant. Tables in the middle show the mean frequency in percentage of cytokine-producing CD4^+^ and CD8^+^ T cells responding to relevant peptides in COPD and healthy participants. Graphs on the right depict the frequency of IFN-γ- and TNF-α-producing CD4^+^ and CD8^+^ T cells, represented as box plots. All individual values are also represented. Red data points represent COPD patients (*n* = 23) and blue data points represent healthy subjects (*n* = 21). IFN-γ^+^ and TNF-α^+^ T cell populations were compared between COPD patients and healthy controls using two-tailed Mann–Whitney U and *p*-values are shown.

We also evaluated the effector function of T cells in response to conserved RV-specific epitopes by determining the production of cytokines (IFN-γ, TNF-α, IL-2, IL-4 and IL-10). To this end, PBMCs from participants were stimulated with RV peptide pools and control conditions, as previously indicated, and cytokines were detected through intracellular cytokine staining (ICS) assays (details in Materials and Methods). RV-M4 was used to stimulate CD4^+^ T cells and RV-M8 to stimulate CD8^+^ T cells. Cytokine-producing T cells were obtained after subtracting those from the mock condition. The gating strategy used to identify CD4^+^ and CD8^+^ T cell populations is shown in Figure 5A.

Cytokine-producing T cells were detectable in COPD and healthy participants, although those producing IL-2, IL-4 and IL-10 remained low or minimally detectable (Figure 5B,C). However, a substantial frequency of IFN-γ and TNF-α-producing T cells in response to RV-specific epitopes was detected in both COPD and healthy participants, without statistically significant differences between groups (Figure 5B,C). Nonetheless, TNF-α-producing CD4^+^ T cells tended to be slightly increased in COPD participants, whereas the frequency of IFN-γ-producing CD4^+^ T cells, a hallmark of Th1 polarization, was comparable or tended to be lower than in healthy participants (Figure 5B). These results differ from the enhanced CD4^+^ T cell activation observed in AIM assays (Figure 3). In contrast, COPD patients showed a tendency toward a higher frequency of IFN-γ and TNF-α-producing CD8^+^ T cells (Figure 5C). While statistical significance was not reached, the noted directional shifts warrant investigation in a larger, highly powered cohort.

### 3.5. T Cell Responses to Conserved RV-Specific Epitopes Do Not Correlate with COPD Severity

**Figure 6:**
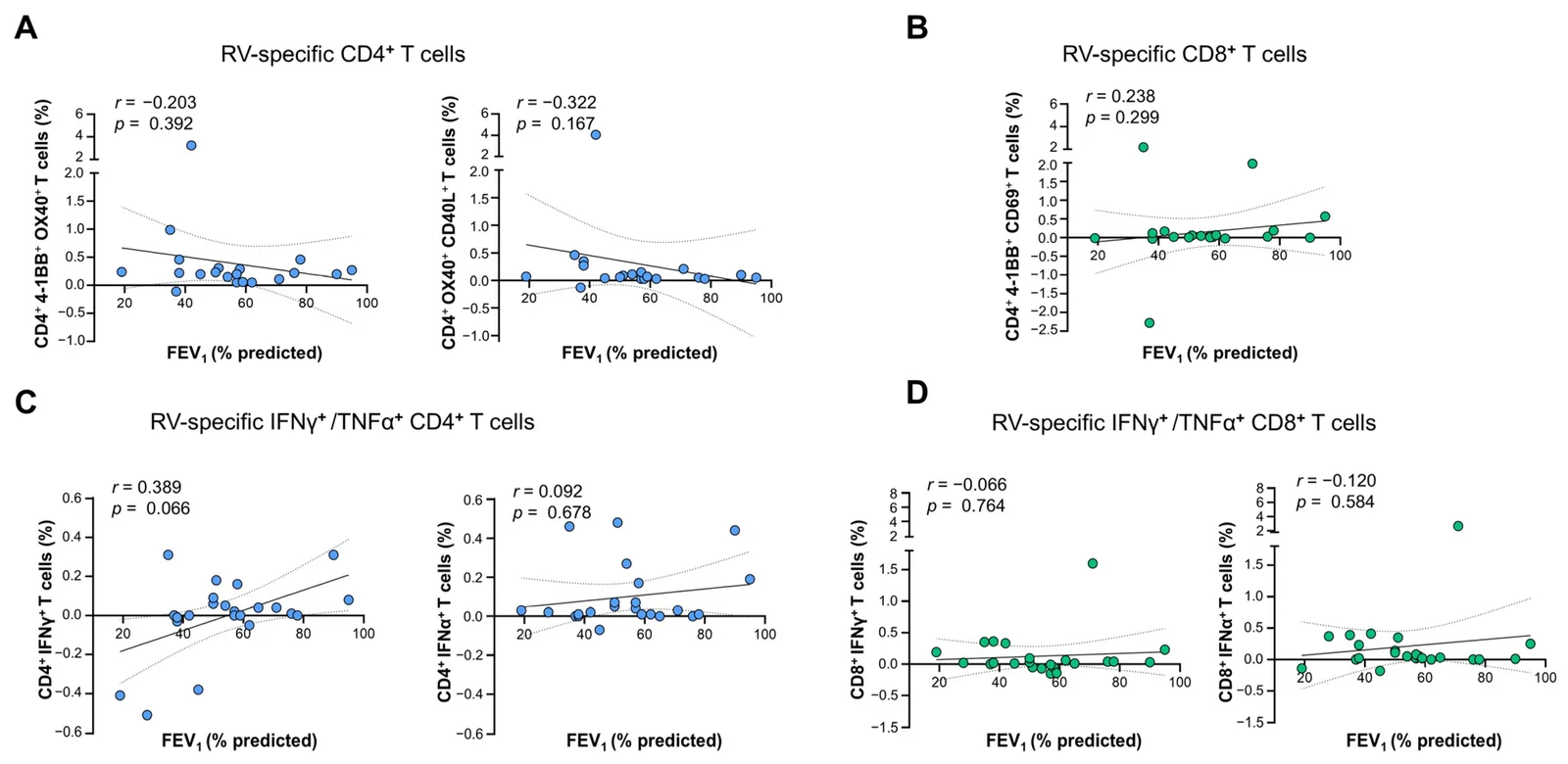
Association between T cell responses to conserved RV-specific epitopes and COPD severity. Spearman’s correlations were carried out to examine the association between RV-specific T cell responses and disease severity, as determined by FEV_1_ (% predicted). (**A**) Spearman’s correlations between the frequency of antigen-specific OX40^+^4-1BB^+^ and OX40^+^CD40L CD4^+^ T cells responding to RV-M4 and FEV_1_ (% predicted) in COPD patients. (**B**) Spearman’s correlation between the frequency of antigen-specific 4-1BB^+^CD69^+^ CD8^+^ T cells responding to RV-M8 and FEV_1_ (% predicted) in COPD patients. (**C**) Spearman’s correlations between the frequency of IFN-γ^+^ and TNF-α^+^ CD4^+^ T cells responding to RV-M4 and FEV_1_ (% predicted) in COPD patients. (**D**) Spearman’s correlations between the frequency of IFN-γ^+^ and TNF-α^+^ CD8^+^ T cells responding to RV-M8 and FEV_1_ (% predicted) in COPD patients. Frequencies of CD4^+^ T cells are represented as blue points, while frequencies of CD8^+^ T cells are represented as green points. The solid and dashed lines depict linear regressions and 95% confidence intervals, respectively. Spearman’s correlation coefficients (*r*) and the corresponding *p*-values are indicated.

Since RV infection is associated with COPD exacerbations, we examined the relationship between T cell responses to conserved RV-specific epitopes and COPD severity. We specifically carried out Spearman’s correlations between the frequency of antigen-specific CD4^+^ and CD8^+^ T cells responding to RV-specific epitopes and FEV_1_ (% predicted). Similarly, we also assessed correlations between IFN-γ and TNF-α-producing CD4^+^ and CD8^+^ T cells with FEV_1_ (% predicted). It is worth noting that the correlation between FEV_1_ (% predicted) and disease severity is inverse. The lower the FEV_1_, the greater the disease severity. As shown in Figure 6, no statistically significant correlations were observed. However, we noticed some interesting trends. Thus, Spearman’s correlation coefficients between the frequency of distinct antigen-specific CD4^+^ T cells and FEV_1_ are negative (~−0.2) (Figure 6A), indicative of a potential small correlation with disease severity. In contrast, Spearman’s correlation coefficient between antigen-specific CD8^+^ T cells and FEV_1_ is positive (*r* = 0.238) (Figure 6B), suggesting a potential small inverse correlation between disease severity and the frequency of antigen-specific CD8^+^ T cells responding to the conserved RV-specific epitopes. Although statistical significance was not reached, these correlations warrant investigation in larger, highly powered cohorts.

The potential relationships between the frequency of cytokine-producing T cells and FEV_1_ are also worth noting. As shown in Figure 6C, there was a modest correlation between IFN-γ-producing CD4^+^ T cells and FEV_1_ (*r* = 0.389), but this did not reach statistical significance (*p* = 0.066), suggesting that stronger Th1 responses to the selected RV-specific epitopes may be associated with better pulmonary function and lower disease severity. However, this potential association requires to be confirmed in a larger, highly powered cohort. In contrast, the correlation between TNF-α-producing CD4^+^ T cells responding to conserved RV-specific epitopes and FEV_1_ was negligible (Figure 6C). Likewise, no remarkable correlation between TNF-α-producing or IFN-γ-producing CD8^+^ T cells and FEV_1_ was found (Figure 6D).

## 4. Discussion

Viral respiratory infections, especially those caused by rhinoviruses (RVs), are major drivers of COPD exacerbations and disease progression [14,15,16]. However, the mechanism is not completely understood. Antiviral innate immunity in COPD patients has been shown to be impaired, which results in greater susceptibility to viral infections, increased viral replication and prolonged antigen exposure [28,29,30]. In parallel, repeated RV infections lead to the accumulation of antigen-specific T cells in the lungs of COPD patients [33], where antiviral Th1/Tc1 responses can contribute to increased airway inflammation [47], thus supporting an enhanced Th1 reactivity in classical COPD [3]. However, anti-RV T cell immunity is also necessary to clear the virus and reduce its pathological impact. Therefore, the role of antiviral/anti-RV T cell immunity in inducing protection or causing disease progression in COPD is unclear and evidence from T cells responding to RV-specific epitopes is missing.

To address this knowledge gap, we carried out an exploratory study aimed at comparing RV-specific T cell responses in COPD and healthy participants to peptide pools, RV-M4 and RV-M8, consisting of experimentally defined CD4^+^ and CD8^+^ T cell epitopes, respectively, conserved in RV-A or RV-C serotypes (Supplementary Table S1). CD4^+^ and CD8^+^ T cell responses were studied *in vitro* using recall stimulations of peripheral blood mononuclear cells (PBMCs) with the relevant peptide pools. Conserved RV-specific T cell epitopes were selected for exhibiting promiscuous binding to HLA molecules and for inducing detectable T cell responses in most people [37,38]. The study cohort included 23 COPD patients and 21 age-matched healthy controls, with a balanced Body Mass Index (BMI) distribution (Table 1). Sex was balanced in healthy participants, but COPD participants were mostly women (*n* = 18). Although COPD is more prevalent in women [48], the female sex bias in the COPD cohort is a limitation of the study (Table 1). The small sample size limits the power of the statistical analysis and the reduced number of males with COPD may limit the generalizability of the findings. COPD participants exhibited substantial heterogeneity in their clinical characteristics. However, most participants were classified as GOLD 2, exhibited moderately reduced DLCO (% predicted), and had blood eosinophil counts between 100 and 300 cells/µL. Moreover, a substantial minority of COPD participants (30.4%) had blood eosinophil counts exceeding 300 cells/µL, compatible with an eosinophilic COPD phenotype [6,7]. FEV_1_ (% predicted) and DLCO (% predicted) correlated weakly (*r* = 0.380; Figure 1B). However, this correlation did not reach statistical significance (*p* = 0.073). Baseline immune status of COPD and healthy participants, as defined by the frequency of distinct lymphocyte populations in unstimulated PBMCs, was comparable. However, differences between COPD and healthy controls became apparent upon stimulation with RV peptide pools.

Stimulation of PBMCs with RV-specific epitope peptides induced a substantial activation of responding CD8^+^ and CD4^+^ T cells in both COPD and healthy participants, as determined in AIM experiments (Figure 3). Moreover, CD4^+^ T cell activation induced by RV-specific epitopes (RV-M4) was significantly stronger in COPD participants than in healthy controls, as evidenced by the increased frequency of different CD69^+^ CD4^+^ T cell subsets (Figure 3B). The frequency of antigen-specific CD4^+^ T cells, characterized by the co-expression of CD40L and OX40, or OX40 and 4-1BB [44,45], was also significantly increased in COPD patients (Figure 3B). The frequency of activated CD8^+^ T cell populations, including antigen-specific CD8^+^ T cells, defined by the co-expression of 4-1BB and CD69 (53), was also elevated in COPD patients compared with healthy controls, but without reaching statistical significance (Figure 3C). In general, CD8^+^ T cell numbers are lower than those of CD4^+^ T cells and activation of CD8^+^ T cells may require some Th help, which cannot be efficiently provided using CD8 epitopes. Hence, detection of statistically significant differences in the activation of CD8^+^ T cells will require carrying out the analysis in a larger, highly powered cohort. However, a correlation between the frequency of antigen-specific CD4^+^ and CD8^+^ T cells responding to RV-specific epitopes was found in COPD patients (Figure 4A), suggesting a heightened coordinated antiviral immune activation. This correlation was not observed in healthy participants (Figure 4B). Given the short stimulation times and the use of defined RV-specific epitopes, the responding T cells likely represent pre-existing antigen-experienced T cells elicited by prior RV infections. Hence, these results support that COPD patients sustain more recurrent RV infections, likely linked to the increased viral susceptibility described in COPD [28,29,30].

We also evaluated the functional profile of RV-specific T cells by intracellular cytokine staining (ICS) (Figure 5). IL-2, IL-4 and IL-10 cytokine production was low in both COPD patients and healthy individuals. However, substantial IFN-γ and TNF-α responses to the selected RV-specific epitopes were detected in both COPD and healthy participants, consistent with pre-existing RV-specific Th1 immunity, with no significant differences between groups (Figure 5B,C). However, a small tendency towards an increase in the frequency of responding TNF-α-producing cells was observed in COPD patients compared with healthy participants in both CD4^+^ and CD8^+^ T cells. Similarly, the frequency of responding IFN-γ^+^ CD8^+^ T cells also tended to be enhanced in COPD participants. In contrast, the frequency of responding IFN-γ^+^ CD4^+^ T cells was comparable or tended to be slightly higher in healthy controls than COPD patients. Therefore, the increased frequency of antigen-specific CD4^+^ T cells detected in COPD patients responding to RV-specific epitopes was not coupled with a concomitant increase in functional cytokine responses, suggesting a degree of functional dissociation between antigen recognition and effector function. This dissociation may point to some level of T cell dysfunction or exhaustion. Indeed, features consistent with T cell exhaustion, including increased expression of inhibitory receptors such as PD-1 and impaired effector function, have been described in COPD, particularly in lung-resident CD8^+^ T cells [31,32]. These features of T cell exhaustion can be the consequence of chronic or repeated antigen exposure rather than an intrinsic defect in T cell function, in agreement with impaired innate antiviral immunity and susceptibility to viral infections in COPD [28,29,30]. However, we studied circulating T cells in PBMCs, which may display a different functional profile than T cells present in the lung parenchyma and did not evaluate T cell exhaustion markers such as PD-1 or TIM-3. Therefore, the disparity between antigen-specific activated T cells and cytokine production might also be explained by alternative physiological phenomena, such as transient T cell anergy due to recent *in vivo* viral exposures or partial tolerization of memory T cells. Moreover, the differences detected between COPD and healthy participants may also reflect differences in previous rhinovirus exposure, smoking history, cumulative respiratory infections, or other clinical variables that remain to be investigated in larger, highly powered cohorts. Smoking is particularly relevant in COPD, as smoking changes adaptive immunity with persistent effects [49] and contributes to enhanced airway inflammation in COPD [50].

Arguably, the noted degree of functional dissociation between the enhanced frequency of antigen-specific T cells responding to RV-specific epitopes and functional cytokine responses in COPD may also be linked to treatments, in particular to inhaled corticosteroids. Indeed, corticosteroids have been shown to suppress T cell activation, proliferation and cytokine production [51] and most COPD participants received some dosage of inhaled corticosteroids (Table 1). However, it is unclear how corticosteroids would suppress cytokine production but not T cell activation, which was enhanced in COPD participants. Moreover, while it has been reported that airway proinflammatory cytokines decrease in COPD smokers receiving inhaled corticosteroids [52], it has also been shown that IFN-γ production from COPD airway lymphocytes is corticosteroid insensitive [53]. Whether this insensitivity extends to circulating lymphocytes in PBMCs remains unexplored. Therefore, a potential confounding effect of corticosteroids in antigen-specific responses warrants further exploration in a larger cohort, enabling appropriately powered stratification concerning corticosteroid usage. Differences in HLA expression between COPD and healthy controls also deserve exploration, as they influence T cell immunity.

Regardless of the potential degree of dysfunction in RV-specific CD4^+^ T cells, functional CD4^+^ T cell responses to the selected RV-specific epitopes were largely preserved in COPD, as cytokine production was overall comparable with healthy controls. Moreover, since Th1/Tc1 cytokine responses to the conserved RV-specific T cell epitopes were not markedly enhanced in COPD patients, their contribution to the immunopathology of COPD exacerbations is questionable and may instead reflect antiviral responses that contribute to viral clearance. Indeed, T cell responses to RV-specific epitopes did not correlate with COPD severity. We instead observed a modest inverse correlation between IFN-γ-producing CD4^+^ T cells responding to conserved RV-specific epitopes and disease severity (lower FEV_1_ values), although it did not reach statistical significance (*p* = 0.066) (Figure 6C). Hence, this exploratory observation requires confirmation in a larger, highly powered cohort. Regardless, airway immunopathology associated with RV-induced COPD exacerbations can occur without requiring exaggerated Th1/Tc1 responses as a result of epithelial and innate immune activation, inflammatory cell recruitment and increased viral replication associated with impaired antiviral immunity [28,29,30].

Overall, our findings suggest that exaggerated RV-specific Th1 responses to conserved epitopes do not substantially contribute to the immunopathology of COPD exacerbations. However, we cannot discard the possibility that anti-RV T cell immunity may be altered in COPD patients. Moreover, T cell responses to viral infections, including RV, are strongly influenced by the specific epitopes recognized. Studies across multiple viral systems have shown that distinct epitopes can elicit qualitatively different T cell responses [54]. Therefore, alternative epitope selections may produce different immune profiles and potentially lead to different conclusions. Indeed, RV proteinase 2A has been reported to induce both Th1- and Th2-associated responses in COPD patients [55]. Notably, none of the peptides evaluated in the present study were derived from proteinase 2A. However, it is worth noting that, due to technical limitations, we did not carry out a comprehensive characterization of functional RV-specific T cell responses. In particular, we did not analyze Th17 responses, characterized by the expression of IL-17A, which can also drive inflammation in COPD [4,5]. Similarly, we did not analyze IL-5 and IL-13, which are relevant in eosinophilic COPD [6,7]. Nevertheless, the observation that COPD patients exhibited enhanced antigen-specific T cell responses to the selected conserved RV-specific epitopes, while maintaining Th1/Tc1 cytokine responses comparable to those of healthy subjects, highlights the potential relevance of these epitopes for vaccine development in COPD.

Currently, there is no approved vaccine against RV. The extensive antigenic diversity of RVs has represented a major obstacle to the development of classical vaccines aimed at inducing broadly protective antibody responses [56,57,58]. Indeed, antibodies induced by RV infection generally exhibit limited cross-serotype activity [34,59]. An alternative and promising strategy for RV vaccine development may be the induction of T cell immunity targeting conserved RV-specific epitopes [60]. T cell-mediated immunity plays a central role in the control of viral respiratory infections and the development of long-term protective immunity. Moreover, T cells have been shown to respond to multiple RV serotypes [61], supporting the recognition of epitopes conserved among diverse RV strains [37,38]. Furthermore, unlike conventional vaccine approaches, epitope-based strategies allow the selective targeting of defined antigenic regions, enabling the induction or enhancement of immune responses directed toward epitopes with desired functional properties while potentially minimizing immunopathology [62]. This feature may be particularly advantageous in COPD, where excessive Th1 responses may play a role in worsening disease severity and exacerbations. Thus, an epitope-based vaccine incorporating the conserved RV-specific epitopes used in this study could hypothetically promote CD8^+^ T cell responses together with controlled Th1 responses, potentially helping to balance antiviral activity with a more regulated inflammatory profile. However, this hypothesis remains to be formally demonstrated, since the present study did not evaluate vaccination, protective immunity or viral clearance.

## 5. Conclusions and Limitations

COPD patients exhibited enhanced frequencies of antigen-specific CD4^+^ T cells recognizing conserved RV-specific epitopes compared with healthy controls while Th1/Tc1 cytokine responses were comparable. Moreover, RV-specific T cell responses did not correlate with disease severity. Collectively, our findings support the notion that RV infections may contribute to the immunopathology of COPD exacerbations through mechanisms beyond merely exaggerated virus-specific Th1 responses. The ability of the selected epitopes to engage antigen-experienced CD4^+^ T cells without promoting an exaggerated Th1 cytokine profile, while maintaining measurable CD8^+^ T cell responses, supports further consideration and research for epitope-based vaccine development in COPD.

This study has several limitations. The use of peptide pools with selections of conserved RV-specific T cell epitopes, while valuable for mechanistic interrogation, does not capture the full repertoire of RV-specific T cell immunity. The sample size was exploratory, appropriate to provide foundational insights into the role of RV-specific T cell immunity in COPD, and sex distribution was unbalanced, with a higher proportion of female COPD participants. The small sample size and gender imbalance limit the statistical power and generalizability of our analysis. Therefore, tendencies and conclusions should be confirmed in larger and more sex-balanced cohorts, allowing clinical stratifications, as additional factors such as treatments, smoking history, HLA and other respiratory infections may also influence RV-specific immune responses, acting as confounding factors. The study was cross-sectional, the participants in the study were not HLA typed and multiple statistical comparisons were not carried out. The flow cytometry instrumentation did not facilitate a comprehensive characterization of antigen-specific T cell responses. Accordingly, we did not include antibodies to analyze the expression of relevant cytokines such as IL-17, IL-5 and IL-13. Future studies using spectral flow cytometry will allow a broader characterization of RV-specific T cell responses. Finally, our analyses are focused on systemic RV-specific responses involving circulating T cells in PBMCs, which do not directly reflect the local immunity in the affected COPD airway. Indeed, mucosal immunity to RV-specific epitopes, including that in the lower and upper airways, may differ substantially and warrants future investigation.

## Figures and Tables

**Table 1:** Characteristics of the cohort.

	COPD Patients *n* = 23			Healthy Participants *n* = 21		
**Gender, *n* (%)**						
Male	5 (21.70%)			9 (42.85%)		
Female	18 (78.20%)			12 (57.14%)		
**Age, Median (Interquartile Range, IQR)**						
All	65.00 (10.50)			62.00 (11.00)		
Male	74.00 (2.00)			61.00 (17.00)		
Female	65.00 (8.50)			63.50 (12.00)		
**BMI ^1^ (kg/m^2^) Median (IQR)**						
All	25.00 (4.00)			26.00 (2.00)		
Male	28.00 (4.00)			27.00 (2.00)		
Female	25.00 (3.50)			25.50 (2.25)		
**Tobacco**						
Active smokers, *n* (%)	4 (17.40%)			1 (4.76%)		
Ex-smokers, *n* (%)	19 (82.60%)			NA ^2^		
Pack-years, Median (IQR)	50.00 (20.00)			NA		
**Clinical parameters**						
FVC mL ^3^, Median (IQR)	2460.00 (545.00)			NA		
FVC % ^4^, Median (IQR)	96.00 (32.00)			NA		
FEV_1_^5^ mL, Median (IQR)	1020.00 (530.00)			NA		
FEV_1_% ^6^, Median (IQR)	54.00 (23.50)			NA		
FEV_1_/FVC %, Median (IQR)	46.05 (15.13)			NA		
DLCO % ^7^, Median (IQR)	45.00 (20.00)			NA		
Eosinophils/µL, Median (IQR)	200.00 (135.00)			NA		
Eosinophils %, Median (IQR)	2.00 (1.14)			NA		
Dyspnea (mMRC ^8^), *n* (%)						
Dyspnea (0–1 mMRC)	5 (21.74%)			NA		
Dyspnea (≥2 mMRC)	18 (78.26%)			NA		
CAT ^9^ quiz score, Median (IQR)	18.00 (9.50)			NA		
**Exacerbations, *n* (%)**						
Moderate exacerbations previous year (≥1)	14 (60.90%)			NA		
Moderate exacerbations previous year (≥2)	4 (17.40%)			NA		
Severe exacerbations previous year (≥1)	5 (21.70%)			NA		
**Grade GOLD** ^10^**, *n* (%)**						
GOLD 1 (FEV_1_% ≥ 80%)	2 (8.70%)			NA		
GOLD 2 (FEV_1_% 50–79%)	13 (56.50%)			NA		
GOLD 3 (FEV_1_% 30–49%)	6 (26.10%)			NA		
GOLD 4 (FEV_1_% < 30%)	2 (8.70%)			NA		
**Inhaled Therapies, *n* (%)**						
LAMA ^11^ or LABA ^12^	1 (4.34%)			NA		
LAMA-LABA combination	3 (13.04%)			NA		
LABA + ICS ^13^ combination	5 (21.74%)			NA		
Triple Therapy (LAMA + LABA + ICS)	14 (60.87%)			NA		
**ICS therapy dosage, *n* (%)**						
Low dosage	4 (17.30%)			NA		
Moderate dosage	15 (65.21%)			NA		
High dosage	0 (0.00%)			NA		
None	4 (17.39%)			NA		

^1^ BMI (Body Mass Index), ^2^ NA (Data/Information Not Applicable), ^3^ FVC mL (Forced Vital Capacity expressed as mL), ^4^ FVC % (Forced Vital Capacity expressed as % predicted) ^5^ FEV_1_ mL (Force Expiration Volume in 1 s expressed as mL), ^6^ FEV_1_% (Force Expiration Volume in 1 s expressed as % predicted), ^7^ DLCO (Diffusing capacity of the Lungs for Carbon Monoxide), ^8^ mMRC (Modified Medical Research Council Dyspnea Scale), ^9^ COPD Assessment Test (CAT), ^10^ Global Initiative for Chronic Obstructive Lung Disease (GOLD), ^11^ Long-Acting Muscarinic Antagonist (LAMA), ^12^ Long-Acting Beta-Agonist (LABA) and ^13^ Inhaled Corticosteroids (ICS).

## Data Availability

The original contributions presented in this study are included in the article/Supplementary Material. Further inquiries can be directed to the corresponding authors.

## Supplementary Materials

The following supporting information can be downloaded at https://www.mdpi.com/article/10.3390/biom16101423/s1, Figure S1: Fluorescence minus one (FMO) controls for intracellular cytokine staining assays; Table S1: List of RV-specific CD8^+^ and CD4^+^ T cell epitopes; Table S2: Multiparametric flow cytometry panel used in activation-induced marker (AIM) assays. Table S3: Multiparametric flow cytometry panel used in intracellular cytokine staining (ICS) assays.

## Abbreviations

The following abbreviations are used in this manuscript:

AIM	Activation-induced marker
BMI	Body Mass Index
DLCO	Diffusing capacity of the lungs for carbon monoxide
COPD	Chronic obstructive pulmonary disease
FVC	Forced Vital Capacity expressed
FEV_1_	Forced Expiration Volume in 1 s
GOLD	Global Initiative for Chronic Obstructive Lung Disease
HLA	Human Leukocyte Antigen
ICS	Intracellular cytokine staining
LABA	Long-Acting Beta-Agonist
LAMA	Long-Acting Muscarinic Antagonist
RV	Rhinovirus
