# Supplementary material for: Enhanced Frequency but Preserved Th1/Tc1 Responses of Rhinovirus-Specific T Cells in COPD
Source: Biomolecules. 2026 Oct 1;16(10):1423. doi: 10.3390/biom16101423 (PMC13702297; doi:10.3390/biom16101423)
Supplement: Supplementary file 1 [file biomolecules-16-01423-s001.zip › Supplementary_Figure_S1.pdf]

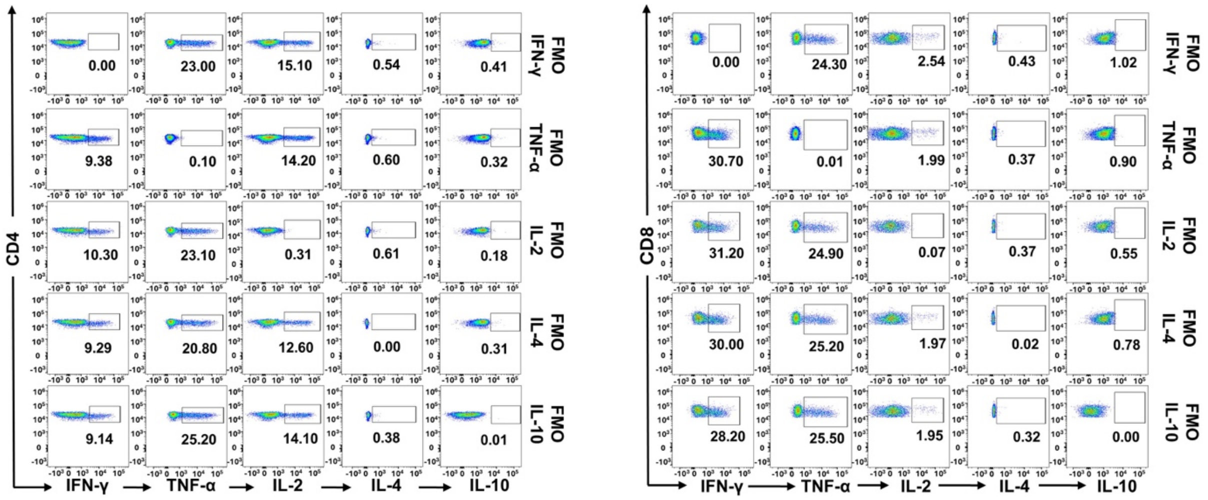

Supplementary Figure S1. Fluorescence minus one (FMO) controls for intracellular cytokine staining assays. FMO controls were used to determine positive expression gates for IFN-γ, TNFα, IL-2, IL-4 and IL-10 in $CD4^+$ and $CD8^+$ T cells. Human PBMCs were incubated for 24 hours (37ºC and 5% CO_2) with T Cell Activation/Expansion Kit, with the addition of brefeldin and monensin (Golgi inhibitors). Cells were stained extracellularly with anti-CD3, CD8 and CD4 antibodies and intracellularly with all anti-cytokine antibodies (anti-IFN-γ, TNF-α, IL-2, IL-4 and IL-10) but the relevant antibody corresponding to the FMO control. Representative dot plots showing FMO controls for IFN-γ, TNF-α, IL-2, IL-4, and IL-10 in $CD4^+$ (left) and $CD8^+$ (right) T cells. The percentages of cytokine-positive $CD4^+$ and $CD8^+$ T cells are indicated. All samples were acquired using a BD FACSCelesta™ flow cytometer (BD Biosciences).
