# Supplementary material for: Enhanced Frequency but Preserved Th1/Tc1 Responses of Rhinovirus-Specific T Cells in COPD
Source: Biomolecules. 2026 Oct 1;16(10):1423. doi: 10.3390/biom16101423 (PMC13702297; doi:10.3390/biom16101423)
Supplement: Supplementary file 1 [file biomolecules-16-01423-s001.zip › Supplementary_Table_S1.pdf]

Supplementary Table S1. List of RV-specific CD8⁺ and CD4⁺ T cell epitopes

Epitope	Sequence	RV Species	Antigen	HLA I/II Restriction/Binding [1]	Peptide Pool
CD8+ T	VLEKGIPTL	RV-A	VP4	A*02:01	RV-M8
	NLIYRNLHL	RV-A	2A	A*02:01	
	QMVSSVTFI	RV-A	3D	A*02:01	
	RTLVLDAYK	RV-A	3D	A*30:01	
	YGDDVIFSY	RV-A	3D	A*01:01	
	ALYIPPYEL	RV-A	3D	A*02:01	
	KYFNINYYK	RV-C	VP4	A*11:01	
	GLEPLDLNTSAGFPYV	RV-C	3D	A*02:01	
	NTSAGFPYV	RV-C	3D	A*02:01	
CD4+ T	VKDVLEKGIPTLQSPTVE	RV-A	VP4	DQA1*01:02/B1*06:02	RV-M4
	DSTITSQDVANAVVGYGV	RV-A	VP4	DRB1*04:04, DRB1*07:01, DQA1*01:02/B1*06:02, DQA1*02:01/B1*02:02, DQA1*03:01/B1*03:01, DQA1*05:01/B1*02:01, DQA1*05:01/B1*03:01,	
	VANAVVGYGVWPHYLTPE	RV-A	VP4	DRB1*08:02, DQA1*01:01/B1*05:01, DQA1*05:01/B1*02:01, DQA1*05:01/B1*03:01	
	INLRTNNSSTIVVPYIN	RV-C	VP2	DRB1*07:01, DRB1*09:01, DRB1*13:02, DQA1*01:02/B1*06:02, DQA1*02:01/B1*02:02	
	KEKFRDIRRFIP	RV-C	3A	DRB1*08:02, DRB1*11:01, DRB1*11:04	
	GLEPLDLNTSAGFPYV	RV-C	3D	DRB1*09:01, DRB1*13:02, DQA1*02:01/B1*02:02	
	DLPYVTYLKDELR	RV-C	3D	DRB1*04:04, DQA1*05:01/B1*02:01	

Table shows the sequence of the rhinovirus (RV)-specific T cell epitopes included in the peptide pools used for *in vitro* stimulation of PBMCs. [1] Confirmed HLA restriction/binding molecules assigned to each epitope based on published experimental data.
