# Supplementary material for: Enhanced Frequency but Preserved Th1/Tc1 Responses of Rhinovirus-Specific T Cells in COPD
Source: Biomolecules. 2026 Oct 1;16(10):1423. doi: 10.3390/biom16101423 (PMC13702297; doi:10.3390/biom16101423)
Supplement: Supplementary file 1 [file biomolecules-16-01423-s001.zip › Supplementary_Table_S2.pdf]

**Supplementary Table S2.** Multiparametric flow cytometry panel used in activation-induced marker (AIM) assays.

| Marker | Fluorochrome | Clone | Provider | Reference |
|---|---|---|---|---|
| CD3 | APC-Cy7 | SK7 | Biolegend | 344818 |
| CD4 | BV605 | SK3 | Biolegend | 344646 |
| CD8 | BV785 | SK1 | Biolegend | 344740 |
| CD154 | PECF594 | 24-31 | Biolegend | 310840 |
| CD69 | BV650 | FN50 | Biolegend | 310934 |
| CD137 | PE | 4B4-1 | Biolegend | 309804 |
| CD134 | APC | Ber-ATC35 | Biolegend | 350008 |
| Live/Dead | Pacific Blue | NA | Thermo Fisher | L34955 |

The multiparametric flow cytometry panel used in AIM experiments consisted of seven different monoclonal antibodies (all from Biolegend) and a viability dye (Live&Dead Pacific Blue, Thermo Fisher Scientific). Compensation was set using CompBeads (BD Biosciences), Arc beads (Thermo Fisher Scientific), according to manufacturer’s instructions, and samples were acquired into a BD Celesta cytometer (BD Biosciences).
