# Supplementary material for: Enhanced Frequency but Preserved Th1/Tc1 Responses of Rhinovirus-Specific T Cells in COPD
Source: Biomolecules. 2026 Oct 1;16(10):1423. doi: 10.3390/biom16101423 (PMC13702297; doi:10.3390/biom16101423)
Supplement: Supplementary file 1 [file biomolecules-16-01423-s001.zip › Supplementary_Table_S3.pdf]

Supplementary Table S3. Multiparametric flow cytometry panel used in intracellular cytokine staining (ICS) assays.

Marker	Fluorochrome	Clone	Provider	Reference
CD3	APC-Cy7	SK7	Biolegend	344818
CD4	BV605	SK3	Biolegend	344646
CD8	BV785	SK1	Biolegend	344740
IFN-γ	BV650	4S.B3	Biolegend	502538
IL-2	PE/CF594	MQ1-17H12	Biolegend	500344
IL-4	BV510	MP4-25D2	Biolegend	500836
IL-10	APC-R700	JES3-9D7	BD Biosciences	567012
TNF-α	AF488	MAb11	Biolegend	502915
Live/Dead	Pacific Blue	NA	Thermo Fisher	L34955

The multiparametric flow cytometry panel used to detect cytokine production in T cells by ICS assays consisted of eight different monoclonal antibodies (Biolegend and BD Biosciences) and a viability dye (Live&Dead Pacific Blue, Thermo Fisher Scientific). For extracellular stains, the panel included monoclonal antibodies against CD3, CD4, CD8, along with a live/dead viability dye. For intracellular stains, the panel included monoclonal antibodies specific for IFN-γ, IL-2, IL-4, IL-10, and TNF-α. Compensation was set using CompBeads (BD Biosciences) and Arc beads (Thermo Fisher Scientific), according to manufacturer's instructions, and samples were acquired into a BD Celesta cytometer (BD Biosciences).
